# Resveratrol Protects against Cerebral Ischemic Injury via Restraining Lipid Peroxidation, Transition Elements, and Toxic Metal Levels, but Enhancing Anti-Oxidant Activity

**DOI:** 10.3390/antiox10101515

**Published:** 2021-09-24

**Authors:** Ming-Cheng Lin, Chien-Chi Liu, Yu-Chen Lin, Chin-Sheng Liao

**Affiliations:** 1 Department of Medical Laboratory Science and Biotechnology, Central Taiwan University of Science and Technology, Taichung 406053, Taiwan; 2 Department of Nursing, National Taichung University of Science and Technology, Taichung 404336, Taiwan; vickyliu@nutc.edu.tw; 3 Department of Medicine, Chung Shan Medical University, Taichung 402306, Taiwan; s0601139@gm.csmu.edu.tw; 4 Laboratory Department, Chung-Kang Branch, Cheng-Ching General Hospital, Taichung 407211, Taiwan; p123765@yahoo.com.tw

**Keywords:** resveratrol, cerebral ischemia, transition element, oxidative stress, toxic metals

## Abstract

Cerebral ischemia is related to increased oxidative stress. Resveratrol displays anti-oxidant and anti-inflammatory properties. The transition elements iron (Fe) and copper (Cu) are indispensable for the brain but overload is deleterious to brain function. Aluminum (Al) and arsenic (As) are toxic metals that seriously threaten brain health. This study was conducted to elucidate the correlation of the neuroprotective mechanism of resveratrol to protect cerebral ischemic damage with modulation of the levels of lipid peroxidation, anti-oxidants, transition elements, and toxic metals. Experimentally, 20 mg/kg of resveratrol was given once daily for 10 days. The cerebral ischemic operation was performed via occlusion of the right common carotid artery together with the right middle cerebral artery for 60 min followed by homogenization of the brain cortex and collection of supernatants for biochemical analysis. In the ligation group, levels of malondialdehyde, Fe, Cu, Al, and As increased but those of the anti-oxidants superoxide dismutase and catalase decreased. Pretreating rats with resveratrol before ischemia significantly reversed these effects. Our findings highlight the association of overload of Fe, Cu, As, and Al with the pathophysiology of cerebral ischemia. In conclusion, resveratrol protects against cerebral ischemic injury via restraining lipid peroxidation, transition elements, and toxic metals, but increasing anti-oxidant activity.

## 1. Introduction

Resveratrol (RVT) is a natural polyphenol compound mainly produced in plants in response to ultraviolet radiation, environmental stresses, injury, and fungal infection [1,2,3,4]. A variety of foodstuffs such as pine nuts, grapes, peanuts, and red wine are rich in the RVT component [1,2,3]. The oral absorption of RVT in humans is about 75% and is thought to occur mainly by trans-epithelial diffusion [1,2,3,4]. Preceding studies have suggested that RVT exhibits a wide range of pharmacological and positively biological activity in animal and human models due to its beneficial effects of anti-inflammation and anti-oxidation [2,3]. Therefore, RVT has been applied not only to effectively eliminate detrimental reactive oxygen species (ROS) but also to up-regulate antioxidant enzyme activity [4,5,6]. Clinically, RVT has been used to attenuate oxidative stress and enhance antioxidant expression in immortalized lymphocytes obtained from Alzheimer’s disease patients [7]. Furthermore, human models have indicated that RVT displays multiple efficacies in scavenging ROS, attenuating lipid peroxidation, and up-regulating anti-oxidant activity so as to effectively ameliorate cerebral ischemic injury, cardiovascular disorders, and metabolic disease [3,5].

Cerebral ischemia is the major disease leading to disability and even death in aged people [8]. Due to its high ROS production, low anti-oxidant capacity, and relatively high component of polyunsaturated fatty acids (PUFA), the brain is vulnerable to oxidative attack [8]. Cerebral ischemic event can generate toxic ROS which can then in turn actively attack the PUFA component of the brain cells, resulting in deleterious oxidative brain injury caused by lipid peroxidation effect [8].

The transition elements of copper (Cu) and iron (Fe) are essential for the brain since they are involved in multiple neurological and brain functions [9,10]. However, excess or deficiency of both elements has been proposed in correlated to some human disorders [11,12,13]. Indeed, due to their actively oxidative property, increased Fe and Cu levels can automatically interact with the hydrogen peroxide via the Fenton reaction and generate toxic hydroxyl radicals (HO^•^). The resulting hydroxyl radicals can then actively oxidize cellular components of PUFA so as to initiate deleterious lipid peroxidation and increase oxidative injury [9,10,11,12]. Therefore, keeping both transition element levels within strict limits helps not only to maintain brain function but also to avoid the threat of oxidative brain injury.

Overload of the toxic metal aluminum (Al) or the metalloid arsenic (As) is considered a serious threat to human health, especially in terms of brain function [14,15,16,17,18]. Previous studies have implicated both substances in the brain in neurological and cerebral disorders [17,18,19]. The toxic mechanism of these hazardous metals in the brain is correlated with ROS generation, lipid peroxidation, and anti-oxidant depletion [13,14,15,16,17]. To date, no studies have detailed the association of the neuroprotective mechanism of RVT during cerebral ischemia with regulation of the concentration of transition metals, hazardous metals, anti-oxidants, or modulation of lipid peroxidation.

## 2. Materials and Methods

### 2.1. Pretreatment of Animal and Harvest of Brain Cortex Samples

In this study, a total of forty Sprague–Dawley male rats which bodyweight ranging from 250 to 300 g were purchased from BioLASCO, the company of laboratory animal breeding and research in Taichung, Taiwan. All rats were kept in stainless-steel mesh cages, housed under controlled conditions (22 ± 2 °C, 50 ± 20% relative humidity, 12-h light-dark cycle) with diet and water for one week in order to stabilize animal conditions followed by randomly separated into four groups as below: control (2% ethanol in the total volume of 1 mL was intraperitoneally given to the rats once in a day for consecutive 10 days); ligation (rats were treated with 2% ethanol once in a day for consecutive 10 days followed by occlusion of the right common carotid artery (RCCA) and right middle cerebral artery (RMCA) for 60 min on day 11); resveratrol (rats were intraperitoneally given with resveratrol at a dosage of 20 mg/kg that is dissolved in 2% ethanol solution once in a day for 10 days); and prevention (rats were pretreated with resveratrol at a dosage of 20 mg/kg that is dissolved in 2% ethanol once in a day for 10 days followed by occlusion the artery of RCCA and RMCA for 60 min on day 11). Experimentally, after 60 min of artery ligation, rats were sacrificed and fresh brain cortex samples were immediately isolated. In order to achieve precisely analytical data, the obtained fresh brain cortex was used to perform the following biochemical analysis including the MDA, transition elements, toxic metals, and antioxidant activity.

### 2.2. Analysis of Malondialdehyde (MDA) Concentration in Brain Cortex Homogenates

Experimentally, 0.2 g of the obtained brain cortex samples were homogenized in the volume of 5 mL of ice KCl (154 mM) by Teflon pestles homogenizers, and the supernatants were immediately harvested. Experimentally, 200 µL of the supernatant was mixed with 3 mL of the H_3_PO_4_ and 800 µL of the KCl solution followed by vortex well. The standard solution of 1, 1, 3, 3-tetra ethoxy propane was used to react with the thiobarbituric acid (TBA) substance and was boiled for 1 h. Finally, 4 mL of the butanol solution was added into the solution and vortex for 5 min followed collected the supernatant for MDA analysis. In general, the detective principle of this present assay relied on the measurement of the pink color that is generated through the reaction of MDA with TBA. The concentrations of brain tissue MDA were determined by spectrophotometer at 532 nm (U-1900, Hitachi, Japan). 

### 2.3. Transition Elements and Toxic Metals Measurement in Brain Cortex Homogenates

In order to determine the levels of Fe, Cu, As, and Al in brain tissue, 2 mL of ultra-pure-grade nitric acid solution was used to wet-digest with 0.2 g of the brain cortex sample overnight. Experimentally, 50% nitric acid solution was prepared for completely soaking all containers that were used to analyze transition and toxic metals. The standard solutions of all measured metals were purchased from Merck (Darmstadt, Germany). The instrument of the SavantAA Z graphite furnace atomic absorption spectrophotometer, which was purchased from the company of GBC Scientific Equipment Pty Ltd. (Braeside, Australia) with longitudinal Zeeman Effect background correction and PAL4000 auto-sampler system experimentally.

### 2.4. Analysis of Enzyme Activity in Brain Cortex Homogenates

Antioxidant activity of SOD was measured based on the procedures of Cayman’s superoxide dismutase assay kit which is purchased from Cayman Chemical Company (Ann Arbor, MI, USA). Basically, xanthine oxidase can react with the hypoxanthine so as to generate the superoxide radical (O_2_^•−^). The superoxide radical has interacted with the tetrazolium salt and the enzyme activity of SOD was measured via the instrument of spectrophotometry (Thermo Scientific Multiskan Spectrum, Vantaa, Finland). The SOD activity was expressed in terms of unit per mg of protein concentration. On the other hand, the CAT levels were assayed by catalase commercial kit purchased from Cayman Chemical Company (Ann Arbor, MI, USA). In brief, this analytical procedure is that the methanol reacts with hydrogen peroxide under the catalyzation of the CAT enzyme so as to generate the formaldehyde. Finally, the chromogen of 4-amino-3-hydrazino-5-mercapto-1, 2, 4-triazole was reacted with the formaldehyde and the CAT levels were measured via spectrophotometer (Thermo Scientific Multiskan Spectrum, Vantaa, Finland), and enzyme activities were expressed in terms of U per mg of protein concentration.

### 2.5. Protein Concentration Analysis in Brain Cortex Homogenates

The commercial kit of BioChain protein assay (San Francisco, CA, USA) was used in the present study. Basically, the principle of this protein assay kit was improved by the method of Coomassie Blue G. Experimentally, the reagent was reacted with the protein and produced a blue color complex and its color intensity is paralleled with protein concentration. The protein concentration was measured via spectrophotometry (Thermo Scientific Multiskan Spectrum, Vantaa, Finland) at the wavelength of 595 nm in the present work.

### 2.6. Data Analysis

The obtained data were expressed as mean ± S.D. The experimental value was analyzed by the Kruskal–Wallis one-way analysis of variance (ANOVA) method. Also, if the obtained data exhibit significant differences among groups, the Fisher’s Least Significant Difference (FLSD) method was used to compare each group. Once the *p*-value was less than 0.05, the statistical differences were considered significantly in the present work. a: *p* < 0.05, vs. control; b: *p* < 0.05, vs. ligation.

## 3. Results

### 3.1. Malondialdehyde (MDA) Concentration in Homogenates of Brain Cortex

**Figure 1:**
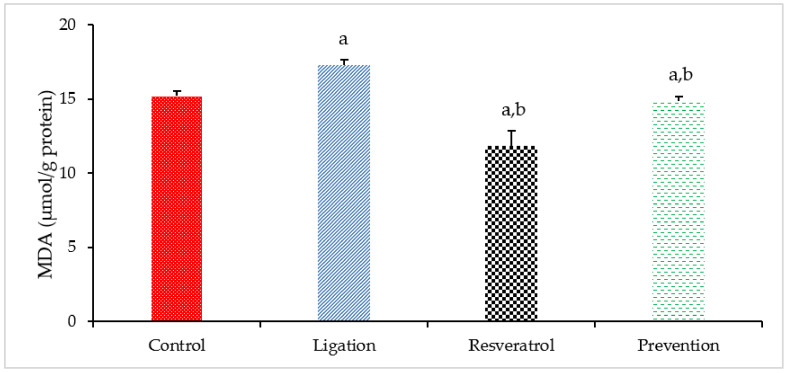
Malondialdehyde (MDA) concentration in the right brain cortex homogenates. Data were expressed as mean ± S.D. The sample number of each group is ten. SD = standard deviation. The Kruskal–Wallis one-way analysis of variance (ANOVA) followed by Fisher’s least significant difference test were used in this study. Difference of statistic was considered significant at *p* < 0.05. a: *p* < 0.05 vs. control subject; b: *p* < 0.05 vs. ligation subject.

The MDA is the end-product of the lipid peroxidation effect and its concentration is paralleled with the intensity of ROS-mediated oxidative injury. As listed in Figure 1, a higher MDA level was found in the ligation group as compared to the control subject. Our result means that cerebral ischemic insult may generate numerous reactive oxygen species (ROS) and these toxic ROS can actively attack the component of poly-unsaturated fatty acid on brain cells so as to promote adversely lipid peroxidation. In terms of the preventive effect, pretreating rats with RVT before ischemic surgery markedly attenuated the MDA level in the ischemic brain cortex as compared to the ligation group. Our result clearly confirms that cerebral ischemia may generate ROS but pretreatment of rats with RVT prior to ischemia obviously eliminates deleterious ROS so the MDA concentration was significantly decreased in the prevention subject.

### 3.2. Transition Elements and Toxic Metal Levels in Brain Cortex Homogenates

**Figure 2:**
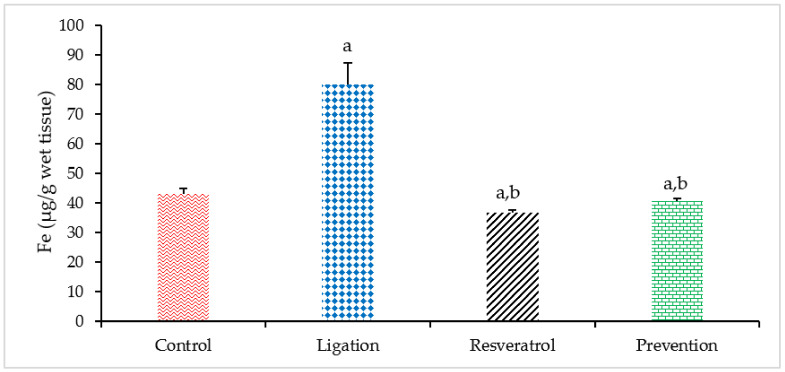
Iron (Fe) level in the right brain cortex homogenates. Data were expressed as mean ± S.D. The sample number of each group is ten. SD = standard deviation. The Kruskal–Wallis one-way analysis of variance (ANOVA) followed by Fisher’s least significant difference test were used in this study. Difference of statistic was considered significant at *p* < 0.05. a: *p* < 0.05 vs. control subject; b: *p* < 0.05 vs. ligation subject.

The iron (Fe) element is essential for brain functions. However, excess of which concentration has been evidenced in associated with human diseases. Regarding the Fe level, a significant increase of the Fe level was found in the ligation subject as compared to the control group (Figure 2). Our result confirmed the fact that cerebral ischemia can result in Fe overload. In terms of the preventive effect, pretreating rats with RVT before ischemic damage obviously diminished the Fe concentration as compared to the ligation group. Our result reveals that RVT can exert its chelating ability in binding with the Fe element so as to the Fe level in the ischemic brain cortex is significantly reduced and importantly, this phenomenon is helpful for attenuating Fe-mediated Fenton reaction and further oxidative brain lesion.

**Figure 3:**
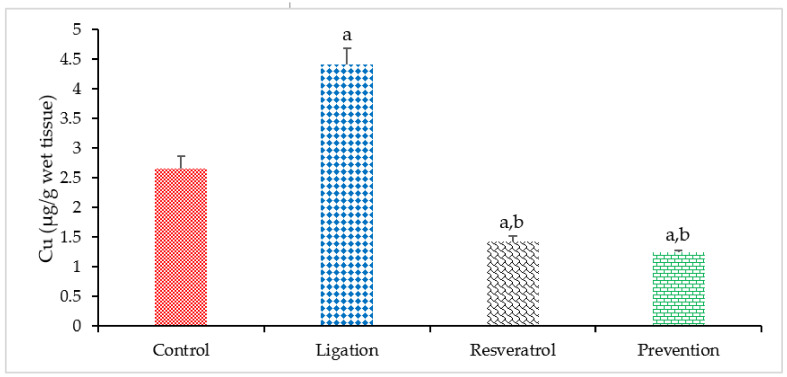
Copper (Cu) level in the right brain cortex homogenates. Data were expressed as mean ± S.D. The sample number of each group is ten. SD = standard deviation. The Kruskal–Wallis one-way analysis of variance (ANOVA) followed by Fisher’s least significant difference test were used in this study. Difference of statistic was considered significant at *p* < 0.05. a: *p* < 0.05 vs. control subject; b: *p* < 0.05 vs. ligation subject.

Regarding the result of the Cu level, excessive Cu level has been documented to result in oxidative tissue injury. Compared to the control group, the Cu concentration was markedly elevated in the ligation subject (Figure 3). It means that ischemic insult can lead to Cu overload. By contrast, pretreating rats with RVT prior to ischemic injury significantly decreased the Cu level in the ischemic brain cortex. Our experimental result reveals that the Cu element is obviously chelated by the RVT so that the Cu concentration is significantly diminished in the ischemic brain cortex. Also, this effect provides by RVT is beneficial to the ischemic brain cortex not only to ameliorate Cu-mediated Fenton reaction but also to decrease further adversely lipid peroxidation effect.

**Figure 4:**
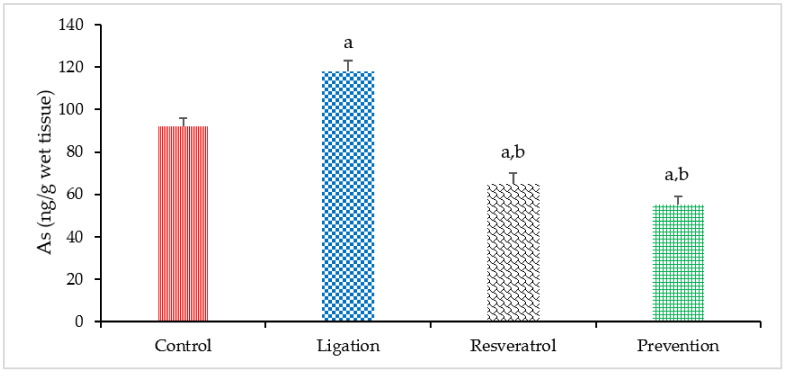
Arsenic (As) level in the right brain cortex homogenates. Data were expressed as the mean ± S.D. The sample number of each group is ten. SD = standard deviation. The Kruskal–Wallis one-way analysis of variance (ANOVA) followed by Fisher’s least significant difference test were used in this study. Difference of statistic was considered significant at *p* < 0.05. a: *p* < 0.05 vs. control subject; b: *p* < 0.05 vs. ligation subject.

As is not required for the brain and is regarded as one of the most toxic metals or metalloids to human health. According to our experimental finding, cerebral ischemia significantly increased the As levels in the ligation subject as compared to the control group as listed in Figure 4. Our result demonstrates that cerebral ischemia can result in As overload. However, pretreatment of animals with RVT before ischemic event prominently reduced the As levels in the prevention subject as compared to the ligation group. Our finding demonstrates that RVT significantly chelates toxic As and that this effect offers by RVT is thinkable to be advantageous in attenuating As-mediated oxidative brain damage.

**Figure 5:**
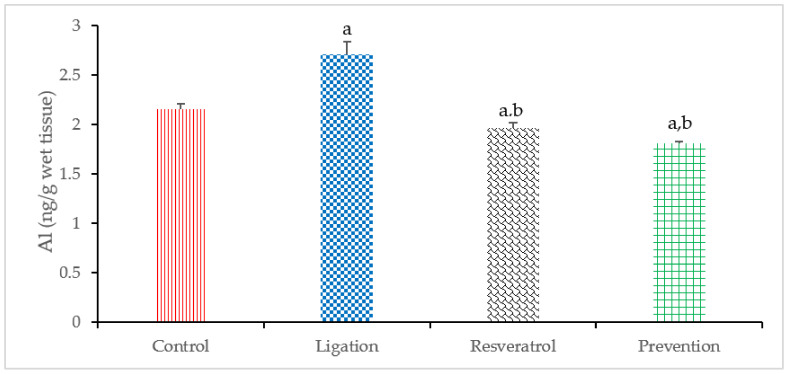
Aluminum (Al) level in the right brain cortex homogenates. Data were expressed as the mean ± S.D. The sample number of each group is ten. SD = standard deviation. The Kruskal–Wallis one-way analysis of variance (ANOVA) followed by Fisher’s least significant difference test were used in this study. Difference of statistic was considered significant at *p* < 0.05. a: *p* < 0.05 vs. control subject; b: *p* < 0.05 vs. ligation subject.

Toxic metal Al is a heavily contaminated metal in the environment and is accumulated mainly in the brain. Meanwhile, the toxic mechanism of Al is implicated with increased oxidative stress which results from ROS generation. In this study, the Al level was obviously high in the ligation subject relative to the control group (Figure 5). This finding indicates that cerebral ischemic insult indeed results in Al overload in the ischemic brain cortex. In terms of the preventive efficacy of RVT, rats administered with RVT before ischemic injury significantly reduced the Al concentration as compared to the ligation group. This phenomenon means that RVT exerts the metal chelating ability to significantly chelate the Al so as to prevent the ischemic brain cortex from further Al-mediated oxidative brain injury.

### 3.3. Antioxidant Enzyme Activity in the Homogenates of Brain Cortex

**Figure 6:**
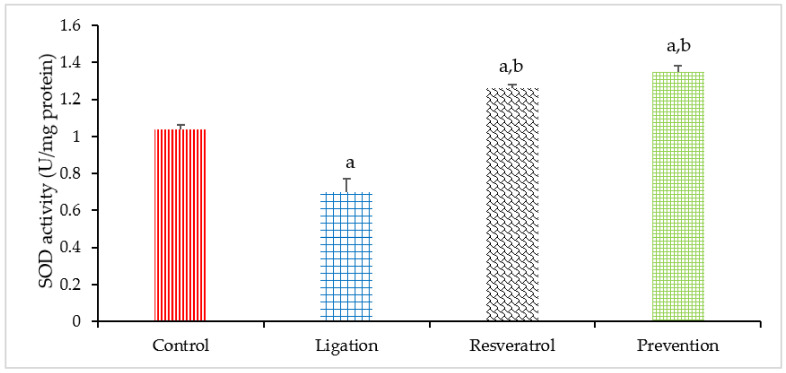
Superoxide dismutase (SOD) activities in the homogenates of right brain cortex. Data were expressed as mean ± S.D. The sample number of each group is ten. SD = standard deviation. The Kruskal–Wallis one-way analysis of variance (ANOVA) followed by Fisher’s least significant difference test were used in this study. Difference of statistic was considered significant at *p* < 0.05. a: *p* < 0.05 vs. control subject; b: *p* < 0.05 vs. ligation subject.

Cerebral ischemic injury can generate toxic superoxide radicals so as to decrease the antioxidant capacity within the cells. In this study, the SOD activity was prominently decreased in the ligation group as compared to the control subject (Figure 6). This result confirms the fact that cerebral ischemia-induced superoxide radicals significantly deplete SOD activity and this effect is thinkable to be disadvantageous to the ischemic brain. In terms of the preventive effect, pretreating rats with RVT prior to ischemic injury significantly enhanced the SOD activity as compared to the ligation subject. This observation suggests that RVT is capable of promoting SOD expression. In this situation, further ROS-mediated oxidative brain damage is as a consequence attenuated.

**Figure 7:**
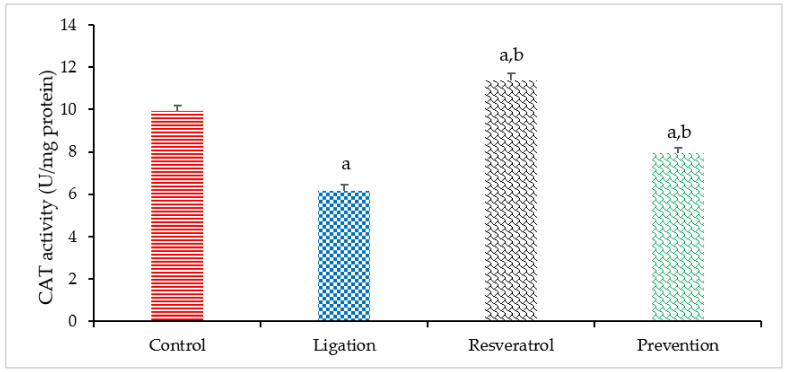
Catalase (CAT) activities in the right brain cortex homogenates. Data were expressed as mean ± S.D. The sample number of each group is ten. SD = standard deviation. The Kruskal–Wallis one-way analysis of variance (ANOVA) followed by Fisher’s Least significant difference test were used in this study. Difference of statistic was considered significant at *p* < 0.05. a: *p* < 0.05 vs. control subject; b: *p* < 0.05 vs. ligation subject.

Proper CAT activity is helpful to eliminate cerebral ischemia-generated toxic hydrogen peroxide. Compared to the control group, a significant decrease in the CAT activity was found in the ligation subject (Figure 7). This result representative that cerebral ischemia indeed generates toxic hydrogen peroxide so as to deplete CAT activity. In terms of the preventive effect of RVT on modulating the CAT, the CAT activity was markedly increased in the prevention group as compared to the ligation subject. Our result reveals that RVT exerts neuroprotective efficacy against cerebral ischemic injury is associated with directly enhancing the CAT activity in the ischemic brain cortex.

## 4. Discussion

Our present findings highlight the correlation of increased levels of transition elements and toxic metals to the pathophysiology of cerebral ischemia. Moreover, the neuroprotective mechanism of RVT against cerebral ischemic injury is correlated with enhancing anti-oxidant activity but restraining lipid peroxidation, transition elements, and toxic metal levels. Cerebral ischemic insult can generate toxic ROS, including superoxide radicals, hydrogen peroxide, and hydroxyl radical. These toxic ROS can actively attack the PUFA structure which is the major component of the brain tissues [8]. In fact, due to its high number of double bond structures, PUFA is the primary target of ROS attack, a harmful effect known as lipid peroxidation. Our present results showed an obviously higher MDA level in the ligation group, but pretreatment of rats with RVT before ischemia significantly attenuated this level. As mentioned above, this phenomenon indicates that RVT can exert its anti-oxidant effect to effectively eliminate ROS levels so as to mitigate ROS-mediated lipid peroxidation effect in ischemic brain.

Studies have shown that RVT exhibits a wide range of pharmacological and biological activity in animal and human models [2,3]. RVT is mainly produced in plants in response to environmental stress, injury, fungal infection, or ultraviolet radiation [3,4]. Due to its polyphenol structure of two aromatic rings with four free hydroxyl groups, RVT has been proposed to exhibit beneficial effects of anti-oxidation and anti-inflammation [3,5]. Human models have indicated that the anti-oxidative property of RVT is helpful in scavenging ROS, attenuating lipid peroxidation, and up-regulating anti-oxidant activity so as to effectively ameliorate cerebral ischemic injury, cardiovascular disorders, and metabolic disease [3,5,20]. An animal model showed that RVT exerts ROS scavenging ability so as to ameliorate ROS-induced lipid peroxidation effect in a traumatic brain injury model [21]. Similarly, as compared to ischemic rats, rats pretreated with RVT before middle cerebral artery ligation had markedly reduced MDA levels [22]. Additionally, RVT significantly restrains lipopolysaccharide- and nitric-oxide-induced ROS production, lipid peroxidation, and neuronal injury in rat brain [23,24]. RVT also can obviously inhibit the expression of the inflammatory factors nuclear factor-kappa B and tumor necrosis factor alpha [25]. Finally, clinical trials in humans have indicated that RVT can inhibit tumor growth, improve cardiovascular disease, modulate energy metabolism, and promote longevity [26]. In the current study, a cerebral ischemic event results in an increased MDA level, but pretreatment of rats with RVT before ischemia markedly attenuated this negative phenomenon. Accordingly, it is crucial to emphasize the fact that RVT exerts a powerful anti-oxidative effect on the affected brain cortex to effectively reduce ROS-mediated lipid peroxidation, and our present result was in agreement with those of former studies.

Transition elements are an integral part of the active site of such macromolecules as proteins, enzymes, and other compositions of the human body [13,27]. Moreover, due to their natural active redox reactions, transition elements can interact with superoxide radicals, hydroxyl radicals, and PUFA so as to generate more ROS within cells [8,9,10,11]. Specifically, transition elements spontaneously interact with toxic hydrogen peroxide through Fenton reaction to generate toxic hydroxyl radicals. The generated hydroxyl radicals spontaneously attack the PUFA macromolecules via lipid peroxidation, resulting in further oxidative cellular damage [8]. Therefore, an increased level of transition elements can promote uncontrolled ROS generation and lead to oxidative damage. As already stressed, it is known that maintaining an appropriate level of transition elements is crucial to brain cells to avoid further oxidative injury. In contrast, increased levels of transition elements have been reported to be associated with the etiology of relevant neurological and cerebral diseases [13,14]. Among these transition elements, attention has predominantly focused on Fe and Cu, mainly due to their indispensable role in cellular functions and their actively oxidative property in nature [11,12,13,14]. Under normal situations, Fe is strictly regulated within cells to keep the amount needed for cell metabolism while avoiding toxic levels so as to prevent further oxidative injury [8]. Moreover, in the situation of absence of Fe catalysts, cerebral ischemia-induced ROS molecule is removed quickly. Instead, once free Fe overloads, it can actively interact with the generated H_2_O_2_ that results from ischemic injury via the Fenton reaction, generating more toxic hydroxyl radicals as a result [8,13,14]. As said before, hydroxyl radicals can actively attack PUFA so as to initiate adverse lipid peroxidation and further oxidative injury. In vivo studies have revealed that alteration and injury as a consequence of Fe overload in the brain is directly related to ROS-mediated lipid peroxidation and is a key mechanism of both Alzheimer’s disease and cerebral ischemia [8,13,14,27]. Similar to Fe, Cu is tightly regulated within cells to avoid toxic levels and prevent oxidative lesions [8,10]. Previous studies have shown that Cu not only actively interacts with H_2_O_2_ via the Fenton reaction, but it can also generate toxic hydroxyl radicals and induce lipid peroxidation [10,12,14]. An in vivo study suggested that the adverse effect of Cu overload not only involves the generation of plaque, soluble oligomers, and lipid peroxidation in the brain but also plays an essential role in the etiology of Alzheimer’s disease and cardiovascular disorders [28]. Further, Cu-mediated beta-amyloid plaque accumulation, intracellular tangles in the brain, and ROS formation are the major hallmarks of Alzheimer’s disease [29]. Collectively, free Cu overload not only generates toxic ROS but also promotes lipid peroxidation and furthers brain diseases. In the present study, cerebral ischemia resulted in free Fe and Cu overload. By contrast, pretreating rats with RVT prior to ischemia effectively reduced Fe and Cu accumulation in the ischemic brain. We suggest that this beneficial phenomenon in decreasing Fe and Cu levels is due to the chelating property of RVT. One clinical study demonstrated that supplementation of RVT to patients on hemodialysis with Fe overload significantly reduced levels of both Fe and lipid peroxidation [30]. Another in vivo study showed that RVT can chelate Cu and scavenge ROS so as to inhibit lipid peroxidation of low-density lipoproteins and avoid cardiovascular disease [31]. Animal research has indicated that RVT can improve the effects of both Cu-induced senescence and Alzheimer’s disease [10,12,13]. As mentioned, the positive effect of RVT in reducing both transition elements of Fe and Cu is due to its polyphenol structure. Therefore, it is of note that pretreatment of rats with RVT before ischemia can markedly attenuate free Fe and Cu overload. In this situation, Fe- and Cu-mediated Fenton reactions and ROS generation are mitigated, significantly restraining further ROS-mediated lipid peroxidation in the ischemic brain.

As is not required for living organisms and is regarded as one of the most toxic metals or metalloids to human health [32]. A previous study proposed that As exposure can result in the production of ROS, including superoxide radicals, hydrogen peroxide, and hydroxyl radicals within cellular systems [33]. These generated ROS not only elevate the level of oxidative stress but also promote lipid peroxidation and deplete anti-oxidant activity [13,14,32]. In vivo experiments indicate that As trioxide-induced cardiac toxicity can be ameliorated by RVT through reducing ROS-mediated oxidative stress [32]. Another in vivo study suggests that supplementation of rats with RVT not only alleviates As retention but also attenuates As-induced lung injury via reducing lipid peroxidation and increasing anti-oxidant activity [34]. Animal research reveals that RVT can effectively protect the feline brain from As-mediated cerebral toxicity [35]. Furthermore, free As overload has been reported in correlation with a variety of human diseases such as stroke, heart disease, diabetes, and cancer [36]. Our present findings indicate that cerebral ischemic injury not only results in As overload and increased MDA levels, but also depletes the anti-oxidant activities of SOD and CAT in ischemic brain cortex. Interestingly, pretreating rats with RVT before an ischemic event statistically reduces As and MDA levels but at the same time obviously enhances the anti-oxidant activities of CAT and SOD in the affected brain. Studies provide evidence that the mechanism of As metabolism operates in an energy-dependent manner [37]. Another study has proposed that, once cerebral ischemia occurs, blood flow is unable to enter the ischemic brain [8]. Thereby, poor energy supplementation may exactly interfere with As metabolism. Under this situation, As is accumulated in the ischemic brain. In contrast, a beneficial effect of RVT is that pretreatment of animals with RVT before ischemia not only effectively reduces As and MDA levels, but also enhances the anti-oxidant activities of CAT and SOD in the ischemic brain. A number of in vivo and in vitro studies have illustrated that RVT can significantly enhance SOD and CAT activity [4,5,7]. Increased enzyme activity is helpful to enable the ischemic brain to scavenge toxic ROS and further ROS-mediated lipid peroxidation. Accordingly, the interpretation of our findings is that RVT not only effectively reduces free As levels but also enhances anti-oxidant activity. As a result, As-mediated oxidative stress is reduced in the ischemic brain cortex in the present study. This is the first paper elucidating the neuroprotective mechanism of RVT in modulating As levels during cerebral ischemia.

**Figure 8:**
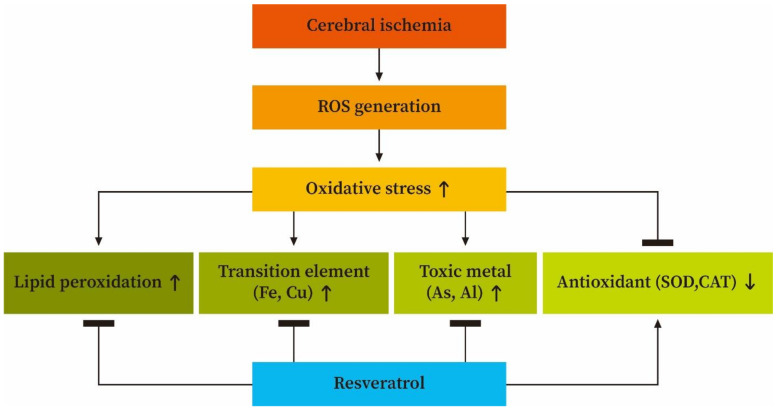
Schematic illustration of the neuroprotective mechanism of resveratrol in modulating the levels of lipid peroxidation, transition elements, toxic metals, and anti-oxidants during cerebral ischemia. ROS, reactive oxygen species.

A huge number of papers reveal that Al is a heavily contaminated metal in the environmental system and is accumulated mainly in the brain [33,38]. Meanwhile, a variety of brain disorders in humans such as Parkinson’s disease, Down’s syndrome, and Alzheimer’s disease have been reported to be correlated with Al overload [33,37,38]. Studies suggest that toxic mechanism of Al is implicated with increased oxidative stress which results from ROS generation combined with depletion of the anti-oxidant capacity [37,38,39]. As mentioned, cerebral ischemic insult can generate a large amount of superoxide radicals in the affected brain. In such a situation, free Al can actively bind with superoxide radicals to generate a variety of toxic ROS such as hydrogen peroxide and hydroxyl radicals [33,38]. When this happens, numerous ROS can spontaneously attack the PUFA component of the ischemic brain cells so as to induce a deleterious lipid peroxidation effect. Animal studies suggests that chronic exposure to Al not only enhances lipid peroxidation and enzyme depletion but also results in impairment of mitochondrial energy metabolism in different brain regions [37,38,39]. Other experimental animal models suggest that neuronal injury via oxidative stress in the cerebral hemisphere of rats exposed to Al is significantly increased in the effect of lipid peroxidation, along with a significant decrease in the anti-oxidant activity of SOD and CAT. As stressed above, it is clear that Al toxicity is directly correlated with ROS generation together with anti-oxidant activity depletion. In contrast, a number of investigations have recently focused on the beneficial neuroprotective effects of RVT [7,40,41]. Previous investigation indicated that RVT attenuates oxidative stress but enhances anti-oxidant expression and anti-aging genes in the immortalized lymphocytes obtained from Alzheimer’s disease patients [7]. Another study suggested that RVT supplementation results in protective activity in the cell-based model of brain ischemia [42]. Additionally, beta-amyloid toxicity in the brain can be attenuated by treatment with RVT [43]. Other experiments in a rat model indicate that treatment with RVT immediately after traumatic brain injury significantly reduces oxidative stress and lesion volume by reducing the levels of MDA and ROS, but enhancing the glutathione activity [44]. Finally, an animal study demonstrated that RVT can effectively prevent Al-induced neurotoxicity via reducing the MDA level but enhancing the activity of SOD and glutathione peroxidase in cerebral tissue [41]. Preceding study indicates that metabolism of the transition and toxic metals within the cells are in an energy-dependent manner [37,38,39]. Cerebral ischemia results in the interruption of energy supply to the affected brain tissues. Under this situation, both transition and toxic metals are accumulated in the ischemic brain cortex as a consequence. Taken together, our present findings highlight that cerebral ischemic injury may result in free Al overload but, interestingly, pretreating rats with RVT before ischemia significantly reduces Al concentrations in the affected brain cortex. We suggest that the mechanism of the neuroprotective action of RVT in reducing free Al levels in the ischemic brain cortex is due to the ability of its polyphenol compound to chelate the Al in advance so as to reduce Al retention. In this situation, reducing Al levels both attenuates ROS generation, so as to effectively diminish ROS-mediated lipid peroxidation, and simultaneously enhances anti-oxidant activity in the ischemic brain cortex. Figure 8 illustrates the proposed neuroprotective mechanism of RVT in modulating the levels of lipid peroxidation, transition elements, toxic metals, and anti-oxidants during cerebral ischemic injury.

## 5. Conclusions

To our knowledge, this is the first paper to highlight how RVT mitigates oxidative injury to the ischemic brain via modulating toxic metals, transition elements, lipid peroxidation, and anti-oxidants. In conclusion, it is important to note that not only can RVT be used for anti-oxidant therapy to prevent or even treat ROS-mediated cerebral disorders, but it can also act as a useful chelating agent for other deleterious transition elements, to diminish toxic metal-induced brain injury resulting in disorders such as neurodegenerative diseases, stroke, cardiovascular diseases, and a variety of ROS-mediated human diseases. Finally, based on its antioxidant property, RVT possesses the useful potential in a variety of fields such as food industry and pharmacology in the future.

## Data Availability

The data presented in this study are available in this manuscript.
